# Epidemiology, Risk Factors, and Perinatal Outcomes of Placental Abruption—Detailed Annual Data and Clinical Perspectives from Polish Tertiary Center

**DOI:** 10.3390/ijerph19095148

**Published:** 2022-04-23

**Authors:** Monika Bączkowska, Katarzyna Kosińska-Kaczyńska, Magdalena Zgliczyńska, Robert Brawura-Biskupski-Samaha, Beata Rebizant, Michał Ciebiera

**Affiliations:** Center of Postgraduate Medical Education, Second Department of Obstetrics and Gynecology, 01-813 Warsaw, Poland; monika.baczkowski@gmail.com (M.B.); katarzyna.kosinska-kaczynska@cmkp.edu.pl (K.K.-K.); zgliczynska.magda@gmail.com (M.Z.); robertsamaha@gmail.com (R.B.-B.-S.); beata.rebizant@gmail.com (B.R.)

**Keywords:** placenta, placental abruption, postpartum hemorrhage, pre-eclampsia, pregnancy outcome, risk factors

## Abstract

Placental abruption (PA) is a separation of the placenta from the uterine wall occurring with the fetus still present in the uterine cavity. It contributes to numerous neonatal and maternal complications, increasing morbidity and mortality. We conducted a retrospective study at a tertiary perinatal care center, which included 2210 cases of labor that took place in 2015 with a PA occurrence of 0.7%. No maternal or fetal death during delivery was reported in this period. The identified PA risk factors were uterine malformations, pPROM, placenta previa spectrum, and oligohydramnios. The significant maternal PA complications identified were maternal anemia, uterine rupture, and HELLP syndrome. Preterm delivery occurred significantly more often in the PA group, and the number of weeks of pregnancy and the birth weight at delivery were both significantly lower in the PA group. PA is a relatively rare perinatal complication with very serious consequences, and it still lacks effective prophylaxis and treatment. Despite its rare occurrence, each center should develop a certain strategy for dealing with this pathology or predicting which patients are at risk. Much work is still needed to ensure the proper care of the mother and the baby in this life-threatening condition.

## 1. Introduction

Placental abruption (PA) is commonly defined as a complete or partial separation of the placenta from the uterine wall that takes place after the 20th week of gestation and prior to birth, with the fetus still present in the uterine cavity. Even though the prevalence of PA is low—about 0.4–1% [1,2], this complication is responsible for 10% of perinatal deaths occurring in developed countries [3].

According to the available data, the most common symptoms of PA are abdominal pain (present in 70%), vaginal bleeding (present in 35–80% of cases), uterine contractions or tenderness, and abnormalities in the fetal heart rate (present in 75% of cases) [3,4,5,6]. According to Mei et al., the clinical presentation of PA, especially when connected with abdominal pain, was related to significantly poorer maternal and fetal outcomes [7].

There are numerous reports regarding PA risk factors, but the data are still inconsistent in many aspects. PA in a previous pregnancy, particularly in cases with a history of multiple PAs, is one of the strongest and most unquestionable PA risk factors [8,9]. Numerous studies also confirmed hypertensive disorders of pregnancy to be one of the most important risk factors for PA [9,10,11,12,13]. The maternal medical history of other chronic diseases is also relevant [14,15,16,17], as are several genetic factors [18]. In addition, underweight and both advanced maternal age and adolescent pregnancies were found to be associated with an increased risk of PA [19,20,21]. Most studies confirmed an increased risk of PA in cases of infertility and assisted reproductive technologies [22,23]. Overall, illicit drug use has been confirmed to be an important risk factor for PA, but the data are conflicting in cases in which several substances are used [2,7,11,22,23,24]. Moreover, some complications occurring during pregnancy increase the risk of PA, e.g., polyhydramnios [24] and placenta previa [25], whereas in cases of preterm premature rupture of membranes, the data are inconsistent [26,27]. Generally, any trauma of the uterus, both old (e.g., previous cesarean section) and current (e.g., caused by physical trauma or iatrogenic damage), increases the risk of PA, but the impact of several causes differs, and in some cases, the data are inconsistent [28,29].

Maternal complications connected with PA include bleeding and postpartum hemorrhage with the need for blood or blood substitute treatment, hypovolemic shock, kidney failure, disseminated intravascular coagulation (DIC), peripartum hysterectomy, and death [30,31]. PA also influences maternal long-term prognosis, morbidity, and mortality [32].

PA is also followed by various complications for the newly delivered baby. Most of all, it is connected with more common prematurity and all of its consequences, including a lower Apgar score, lower birth weight, increased morbidity in the neonatal period, prolonged hospital stay, more common admission to the intensive care unit, and, finally, an increased mortality rate [33].

The study was conducted in the Second Department of Obstetrics and Gynecology, Center of Postgraduate Medical Education in Warsaw. It is a multidisciplinary tertiary perinatal medical care center taking care of patients from across the country, as they are referred here from many regional hospitals. The aims of this study were to estimate the clinical characteristics, incidence, and perinatal outcomes of placental abruption in the female population of Warsaw, basing the analysis on the detailed annual records from this institution and exploring the perspectives and directions for possible improvement of the management of this life-threatening condition.

## 2. Materials and Methods

### 2.1. Data Sources

The performed hospital database search involved all cases of labor that took place in 2015. The following data were collected: maternal age, weight, height, gravidity, parity, maternal history, illicit drug use, placental location, gestational age at delivery, mode of delivery, preterm premature rupture of membranes (pPROM) or premature rupture of membranes (PROM) occurrence, and the duration of hospitalization. In addition, basic data on the neonate were collected (the condition after birth and birthweight).

### 2.2. Study Sample

PA cases were identified on the basis of the International Statistical Classification of Disease and Related Health Problems, 10th revision (code O45) (Available online: https://apps.who.int/iris/handle/10665/246208 accessed on 28 February 2022), and confirmed via the detailed analysis of the available data concerning the clinical symptoms: rapidly developing uterine tenderness, abdominal pain, severe vaginal bleeding/hemorrhage and/or fetal distress, and the presence of a retroplacental clot after delivery. In case of doubts concerning the diagnosis, the record was excluded. After excluding cases with missing data, we included the data of 2210 patients, 16 of whom experienced PA. Preterm delivery was diagnosed if labor occurred before 37 weeks of pregnancy. PROM was diagnosed if a rupture of membranes occurred before the beginning of labor and after 37 weeks of pregnancy. pPROM was diagnosed if a rupture of membranes occurred before the beginning of labor and before 37 weeks of pregnancy. Using the available data, it was not possible to differentiate between fetal growth restriction (FGR) and small for gestational age (SGA) fetuses, as all fetuses below the 10th percentile for estimated fetal weight were classified together in the hospital database. The beginning of the labor was defined as regular uterine contractions leading to the ripening and shortening of the cervix.

### 2.3. Statistical Analysis

The normality of the variable distribution was tested with the Shapiro–Wilk test. Most parameters were found to be non-normally distributed. Statistical differences between groups were estimated using the standard Kruskal–Wallis test and Student’s *t*-test. The results are presented as means, standard deviations, and percentages of cases. Significance was accepted at *p* < 0.05. R version 4.1.0 software was used for statistical analysis.

Multiple logistic regression analysis was used to investigate factors related to PA. The following factors were taken into account when performing univariate analyses: the occurrence of pPROM, PROM, placenta previa, hypertensive disorders of pregnancy, diabetes, fertility treatment, anemia, thrombophilia, liver and thyroid diseases, uterine malformations, FGR/SGA, and oligo- and polyhydramnios. All variables with *p* ≤ 0.01 in the univariate analyses were included in the final multifactorial model.

## 3. Results

### 3.1. Group Characteristics

Group characteristics are presented in Table 1. The incidence of PA was 0.7%. In 31% of cases, the diagnosis was confirmed with a histopathological examination. No significant difference occurred between groups regarding parity or maternal age. When the study group and the control group were divided into subgroups according to age (first subgroup: adolescent pregnancies—below 18 years; second subgroup: pregnancies in women aged 18–34 years; third subgroup: pregnancies with advanced maternal age—above 34 years), the incidence of PA appeared to differ between subgroups. There were no patients <18 years old diagnosed with PA. In the second subgroup, the PA incidence was 0.6%, and in the third group, the PA incidence was 1.1%. However, the differences were not statistically significant. There were no cases of chronic hypertension or multiple pregnancies in the study group. In 50% of cases, PA occurred during pregnancy, and in 50% of cases, PA occurred during labor. All PA cases were delivered by cesarean section, while the rate of cesarean section in the control group was 57%. The difference in the mode of delivery was statistically significant.

### 3.2. Placental Abruption Risk Factors

All the results concerning placental abruption risk factors are presented in Table 2.

The frequency of the hypertensive disorders of pregnancy did not significantly differ between the control group and the study group. There were no cases of diabetes, maternal thrombophilia, liver diseases, renal diseases, or thyroid diseases in the PA group. The frequency of the fertility treatment did not significantly differ between the control group and the study group. Uterine malformations occurred more frequently in the PA group, and the difference was significant in this case, with an aOR of 10.541 (95% CI 1.208–92.017). Bicornuate uterus was a uterine malformation reported in cases of PA. In the control group, the reported uterine malformations included double uterus (8 cases), bicornuate uterus (4 cases), unicornuate uterus (1 case), and non-specified uterine malformation (2 cases).

pPROM was significantly more common in the PA group, and the aOR was 5.331 (95% CI 1.329–21.386) in this case. No cases of PROM were noted in the PA group, probably because of the small number of patients in term in this group. Placenta previa spectrum was an important risk factor confirmed in our study, with a significant difference between groups and an aOR of 34.011 (95% CI 6.429–179.934) in this case. Oligohydramnios was more common in the PA group, and the difference was statistically significant, with an aOR of 14.999 (95% CI 1.718–130.920). There were no cases of polyhydramnios in the PA group.

The frequency of the FGR/SGA did not significantly differ between the PA group and the control group.

### 3.3. Maternal Complications

The results concerning maternal outcomes are presented in Table 3. Our study showed that uterine rupture, HELLP, and maternal anemia occurred significantly more frequently in the PA group. Blood loss was significantly higher in the study group. After division by the mode of delivery, the difference in blood loss between the study group and the control group in patients undergoing cesarean section was not significant. When considered together, the mean blood loss was 406 ± 119 mL in patients with anemia, while in patients without diagnosed anemia, the mean blood loss was 321 ± 118 mL. The difference between the groups was significant (*p*-value < 0.001). No cases of uterine atony or subatony were noted in the PA group. The mean duration of hospitalization was longer in the PA group, but the difference was nonsignificant.

### 3.4. Fetal Complications

The results concerning fetal outcomes are presented in Table 3. Our study showed that the age of the pregnancy at delivery was significantly lower in the PA group than in the control group. Moreover, preterm delivery was significantly more common. Neonatal birth weight was significantly lower in the PA group. No cases of fetal loss during delivery were noted in the PA group.

### 3.5. Limitations

The present results should be analyzed with caution. PA is a clinical diagnosis, and precise diagnostic criteria are still unavailable. Therefore, the misclassification or overlooking of several cases cannot be excluded. Moreover, the majority of the collected data were patient-reported and cannot be verified. Therefore, the data concerning several variables may be under- or overestimated. Another important limitation is related to the fact that the study is retrospective and relies on the accuracy and consistency of the individuals gathering and entering the data. Missing data could not be completed in many cases. In addition, there were no cases of some of the complications in the PA group, for example, cases of polyhydramnios. Therefore, these parameters could not be evaluated, even if some evidence in the literature indicates that they could also be risk factors for PA [9,24].

## 4. Discussion

### 4.1. Group Characteristics and Placental Abruption Risk Factors

The calculated PA incidence of 0.7% is consistent with the literature data [3]. In 31% of cases, the diagnosis was confirmed with a histopathological examination. The procedure of the histopathological examination of the placenta is described as a verifying step, confirming the clinically based diagnosis. Nonetheless, it is not performed in every case of PA. Although there were no statistically significant differences between groups in terms of parity, according to the literature, multiparous women are at a higher risk of PA [34].

The difference between the mean maternal age in the PA and the control group appeared not to be statistically significant. In addition, after dividing participants into three subgroups, the differences in PA incidence between age-dependent groups were still not statistically significant. Nonetheless, according to the literature, both advanced maternal age and adolescent pregnancies were found to be associated with an increased risk of PA [19,21]. However, pregnancies at advanced maternal age are an inevitable result of the changes in lifestyle that have taken place in recent years, and adolescent pregnancies are an important target for improvement in the fields of the sexual education, contraception accessibility, and the proper and early commencement of gynecological medical care [35].

The difference between the incidence of hypertensive disorders of pregnancy in the PA group and the control group was not significant. It might be significant in a larger study group, as reported by numerous literature results [9,10,11,12,13]. This confirms the possible common etiopathology of both conditions. According to the literature, patients with confirmed preeclampsia may also develop PA earlier in pregnancy than patients without this issue. Furthermore, the coexistence of PA with preeclampsia is also connected with more severe maternal and neonatal outcomes [36,37]. Hypertensive disorders during pregnancy are also independent risk factors for neonatal prematurity, stillbirth or neonatal death, lower birth weight, and neonatal cerebral palsy in neonates following PA [36,37,38]. According to the literature, the early onset of the disease raises the risk the most [12], while an association with PA was not observed if blood pressure levels were slightly elevated [39]. Moreover, the lack of patient compliance with treatment was confirmed to have an influence on increasing PA incidence [40]. Additionally, the outcomes (and PA incidence) may be improved by the induction of labor compared with expectant management in term cases [41]. Overall, this emphasizes the importance of proper early diagnosis and adequate management in perinatal care. Regular blood pressure measurements are indeed a part of the standards of perinatal care in most countries. In Poland, they are included in the regularly updated nationwide approved Standards of Perinatal Care [42].

Other maternal morbidities also seem relevant to PA incidence. No cases of diabetes, maternal thrombophilia, liver diseases, renal diseases, or thyroid diseases were noted in the PA group in our study. Nonetheless, maternal medical history and its influence on PA incidence have recently been widely investigated in the literature. Autoimmune diseases constitute an important risk factor [43,44]. However, a recently presented meta-analysis by Liu et al. did not confirm this association in cases of antiphospholipid syndrome [16]. Other diseases linked to PA are diabetes [45], hyperthyroidism and hypothyroidism [15,46], liver diseases [47], renal diseases [48], epilepsy [49], migraines [50], asthma [51], and psychiatric disorders [52]. Several studies also confirmed a rise in PA incidence in patients with uncommon diseases, such as fibromyalgia [53] and inflammatory bowel disease [54]. Some studies also identified several genetic factors [18,55] connected with more common PA occurrence. Moreover, inconsistent data were published regarding inherited thrombophilia [14] and being deaf or hard of hearing [56] and their possible influence on PA occurrence. The multiplicity of the conditions connected with PA underlines the complexity of PA etiopathogenesis, with its numerous features remaining obscure.

The matter of infertility and assisted reproductive technologies and their influence on PA incidence is an interesting target. The majority of studies confirmed an increased risk of PA in this case [22,23]. In our study, there was no significant difference in fertility treatment frequency between the PA group and the control group. Moreover, uterine malformations occurred more frequently in the PA group in our study, and the difference was significant in this case, consistent with data published recently [57]. According to the literature, leiomyomas, polycystic ovary syndrome, and endometriosis also increased the risk of PA [17,58].

Moreover, the course of pregnancy has an impact on PA incidence. pPROM was significantly more common in the PA group, but the literature data concerning this subject are inconsistent [26,27]. No case of PROM was noted in the PA group, probably because of the low number of patients in term in this group. Placenta previa spectrum was an important risk factor confirmed in our study (18.75% in the study group and 0.5% in the control group, aOR 34,011, 95% CI 6.429–179.934), and this is consistent with the literature data [25]. Oligohydramnios was significantly more common in the PA group, although this connection has not been fully elucidated in the literature [59].

Any uterine trauma—occurring either before pregnancy, e.g., a previous cesarian section, or during pregnancy, e.g., physical maternal trauma or iatrogenic injury, for example, as a result of perinatal laser treatment or open intrauterine fetal myelomeningocele repair—is suspected to raise the risk of PA. However, the data are inconsistent in cases of open intrauterine fetal myelomeningocele repair [28,29,60].

There were no statistically significant differences in FGR/SGA incidence between the PA group and the control group. Nonetheless, the conclusions should be drawn carefully, as the data concerning this parameter were quite ambiguous. It would be interesting to observe this association in a larger cohort, as both pathologies are connected with the disruption of the placentation process, and in some aspects, they are described as different manifestations of the same pathology [61].

It is worth mentioning illicit drug use, which was a risk factor that could not be evaluated in our study. Increased PA risk associated with smoking and illicit drug use during pregnancy has been confirmed in many studies, especially as it regards cannabis, crack, and opioids, but it has not been confirmed for the use of other drugs [62,63,64]. Recently presented data questioned an association between alcohol drinking and PA occurrence, while concomitant smoking and alcohol consumption was confirmed to multiply PA risk [2,65]. Smoking is more common in groups with low socioeconomic status and seems to be an independent PA risk factor [66]. Regrettably, during the examined period, the data in the hospital database concerning this parameter were available for a minority of cases. Moreover, the incidence of reported illicit drug use was surprisingly low, inconsistent with literature reports [67,68]. This may suggest that women were not willing to admit to drug use during pregnancy, especially during the brief medical interview on admission to the hospital. Such patient practices have previously been described in the literature [69,70]. Therefore, predicting a significant underestimation, we decided not to take this parameter into account. Nonetheless, it underlines the great importance of education programs and the need for increasing awareness concerning illicit drug abuse during pregnancy, which remains insufficient [71].

Regrettably, we also decided not to consider data concerning weight, height, BMI, and gestational weight gain, as they were also only available for a minority of cases. Nonetheless, it is worth mentioning that a recent meta-analysis by Adane et al. (2019) and some other studies have demonstrated that underweight is a significant risk factor for PA, as is gestational weight gain below expected values [20,72]. Conversely, data concerning obesity are inconsistent, as some authors have found that maternal obesity might even have some protective features [20,73]. Interestingly, lower maternal height was also suspected to increase the risk of PA [74]. This all underlines the relevance of education and social programs focusing on healthy lifestyle, proper diet, supplement intake, and physical activity [75].

Noteworthy, air pollution, contaminated water, and dust particles transported from desert areas have been highlighted frequently over the past years and are considered risk factors for PA [76,77]. Furthermore, a connection between rising temperatures and increased risk of PA and stillbirth was observed [78]. Exposure to poor-quality air and water as well as climate changes seem to be important triggers for the premature separation of the placenta, although the mechanism of this phenomenon has not been well elucidated. A recently published Peruvian study also indicated that childhood abuse, intimate partner violence, and exposure to verbal aggression significantly raise the risk of PA, especially when reported together [79]. Moreover, women who had experienced imprisonment were at an increased risk of PA [80]. Interestingly, the association was noted whether they were in prison during pregnancy or not. Furthermore, several authors have reported the influence of heavy physical exertion [81] and exposure to stressful life events on the incidence of PA [82].

### 4.2. Maternal Complications

Maternal PA complications include acute life-threatening events connected with peripartum hemorrhage and its consequences, the risk of peripartum hysterectomy, and even death [30,31]. Uterine rupture and HELLP occurred in our study significantly more frequently in the PA group, consistent with the literature data [83]. Regarding uterine rupture, because it is a quite rare but very dramatic complication, it is worth mentioning the relevant clinical features of the patients’ histories. The first case was a primigravida at 41 weeks of pregnancy without previous uterine surgeries. The patient was diagnosed with a lack of progress of labor during the second stage of labor, caused by the incorrect position of the fetal head toward the birth canal with a suspected fetal birth weight of about 4000 g. During the cesarean section, a longitudinal rupture in the body of the uterus was found. The baby was born in a fair condition with a neonatal weight of 4030 g. The second case was a multiparous woman at 36 weeks of pregnancy with a history of a previous cesarean section and a bicornuate uterus. During the cesarean section performed because of the suspicion of uterine rupture, the patient was diagnosed with the complete separation of the placenta and the rupture of the uterus in the cesarean section scar. The baby was born in a fair condition, with a neonatal weight of 2990 g. As the number of cases is definitely too low to draw any far-reaching conclusions, it is obvious that cases of prolonged labor, uterine anomalies, and previous uterine surgeries require very careful clinical observation and strict supervision during labor.

Maternal anemia was also significantly more common in the PA group, and blood loss was significantly higher in the PA group, which is consistent with literature results [84]. There were no significant differences in blood loss between groups when taking into account only patients after cesarean section. No cases of uterine atony or subatony were noted in the PA group.

It is worth mentioning that, unfortunately, PA consequences go far beyond the perinatal period and have an influence on long-term maternal prognosis. An increased risk of cardiovascular morbidity and mortality was confirmed in numerous local studies and meta-analyses and in a recently published umbrella review [32]. Although the relationship between cardiovascular diseases and PA is not well-understood, this widely documented connection may indicate a shared etiological component of the conditions. Recently presented data by Riihimaki et al. [85] revealed that patients with a history of PA were also at a higher risk of lung cancer and lower risk of breast cancer. They also tended to die younger than women without a history of PA and were at a higher risk of death [86]. The causes of death included more common respiratory tract malignancies, coronary disease, alcohol-related causes, and suicide. This all underlines the need for proper management of such patients, not only in the acute period but also for the rest of their lives.

### 4.3. Fetal Complications

One of the greatest concerns in PA management is the issue of iatrogenic preterm births, which are impossible to avoid but not free of consequences. In our study, the age of pregnancy at delivery was significantly lower in the PA group than in the reference group. Preterm delivery was also significantly more common, which is consistent with literature data [33,36,37]. Neonatal birth weight was significantly lower in the PA group in our study, consistently with literature data [36,37]. Various studies have revealed that PA is also connected with a lower Apgar score at birth, an increased risk of cerebral palsy, neonatal hypoxic–ischemic encephalopathy, intracranial hemorrhage, coagulation dysfunction, breathing difficulties, prolonged hospital stay, and more common admission to the intensive care unit [33,37,38,87]. The severity of neonatal complications is also correlated with the percentage of the prematurely separated placenta [87,88]. No cases of fetal loss during the delivery were noted in our study in the PA group, although numerous authors have reported increased rates of stillbirth and neonatal death in cases of PA [33,36]. Interestingly, according to Riihimaki et al., PA also increased the overall mortality in children, who primarily survived, by 15-fold in the neonatal period (0–27 days) and 10-fold in the first year of life (28–365 days), and this increase remained significant even later [89].

### 4.4. Prevention of Placental Abruption

PA is suspected to be the visible endpoint of processes beginning in the early stage of pregnancy rather than an emergency obstetric complication itself [90]. Although there are no commonly used guidelines to outline the PA risk group, there have already been some attempts to discover first- or second-trimester PA markers. Mothers with the serum levels of the PAPP-A protein < 5th percentile, AFP > 95th percentile, and inhibin-A <5th percentile or >95th percentile were at a higher risk for developing PA later in pregnancy [91]. In addition, early pregnancy serum metabolomic profiles connected with abnormal vaginal bleeding may serve as predictors of PA [92]. Routine second-trimester uterine artery Doppler ultrasound identified about 60% of women at risk of placental complications. However, it failed to improve short-term maternal and neonatal morbidity and mortality [93]. It was shown that daily antepartum low-molecular-weight heparin injections seemed to reduce the risk of PA, although the data were inconsistent [94]. The role of low-molecular-weight heparin prophylaxis for PA deserves further study. Data on PA incidence in women receiving aspirin prophylaxis are also inconsistent and demand further examination [95].

### 4.5. Management of Placental Abruption

The management of PA is still challenging, even for the most experienced obstetricians and perinatologists. In view of this, great emphasis should be placed on first-line attending physicians and midwives, who should filter patients with an increased risk of pregnancy complications. Another direction is implementing the best possible organizational preparation of maternity centers for the quick admission and appropriate treatment of such patients. In our study, thankfully, no maternal loss or fetal death occurred during delivery in the studied PA group. This was partially due to the excellent work of the whole perinatal team and their rapid and adequate actions. One of the most important matters in this case is time. A prolonged decision-to-delivery interval increases perinatal morbidity and mortality [96]. Therefore, there is a substantial need for medical staff training and performing simulations of such challenging situations. This provides faster decision-making, brings knowledge and skills up to date, improves teamwork, and builds self-confidence. This is particularly important because, as it turned out, even in specialized care centers, such situations happen several times a year, making it impossible to provide adequate opportunities for sufficient real-life training for the whole team. A document concerning the standards of perinatal care that seem to have an influence on the improvement of pregnant women’s care, as well as equal access to medical care, is regularly issued and updated in Poland. In fact, as a result of all actions taken, a constant, significant improvement in worldwide perinatal care has been reported by various teams [97,98].

## 5. Conclusions

PA is a relatively rare perinatal complication with very serious short- and long-term consequences. Although we know several risk factors and have a number of hypotheses on PA etiopathogenesis, we do not have any prophylaxis methods or effective treatment. Despite the rare occurrence of PA, every center should develop a strategy for dealing with this pathology or predicting patients who are at risk. Much work is still needed to ensure the proper care of the mother and baby in this life-threatening condition.

## Figures and Tables

**Table 1:** Group characteristics.

		Study Group—Placental Abruption	Control Group	*p*-Value
Number of cases		16	2194	
Maternal age in years (mean ± SD)		32.3 ± 4.7	31.9 ± 4.9	0.8
Subgroups of patients according to age				0.2
	Number of patients <18 years	0	9	
	Number of patients 18–34 years	9	1527	
	Number of patients >34 years	7	658	
Parity (%)				0.9
	Nulliparous	50	47.81	
	Multiparous	50	52.19	
Chronic hypertension (%)		0	1	
Multiple pregnancies (%)		0	1	
Placental abruption during pregnancy (%)		50		
Placental abruption during labor (%)		50		
Mode of delivery (%)				<0.001
	Vaginal delivery	0	43	
	Cesarean section	100	57	

**Table 2:** Placental abruption risk factors.

	Study Group—Placental Abruption	Control Group	*p*-Value	aOR	95% CI	*p*-Value
Preterm premature rupture of membranes (%)	18.75	3.37	<0.001	5.331	1.329–21.386	0.02
Premature rupture of membranes (%)	0	8.34	0.4			
Placenta previa (%)	18.75	0.5	<0.001	34.011	6.429–179.934	<0.001
Hypertensive disorders of pregnancy (%)	6.25	4.28	0.7			
Diabetes (%)	0	5.61	0.3			
Fertility treatment (%)	6.25	3.33	0.5			
Maternal thrombophilia (%)	0	1.09	0.7			
Liver disease (%)	0	0.82	0.7			
Thyroid disease (%)	0	5.38	0.3			
Uterine malformation (%)	6.25	0.68	0.009	10.541	1.208–92.017	0.03
Fetal growth restriction/small for gestational age (%)	6.25	1.46	0.1			
Oligohydramnios (%)	6.25	0.59	0.004	14.999	1.718–130.920	0.01
Polyhydramnios (%)	0	0.32	0.8			

aOR—adjusted odds ratio, 95% CI—95% confidence interval.

**Table 3:** Maternal and fetal outcomes.

		Study Group—Placental Abruption	Control Group	*p*-Value
Age of pregnancy at delivery in completed weeks (mean ± SD)		33 ± 5	39 ± 2	<0.001
Preterm delivery (%)		68.75	9.98	<0.001
Duration of hospitalization in days (mean ± SD)		8.5 ± 7.2	5.4 ± 4.6	0.1
Maternal anemia (%)		25	7.11	0.006
Blood loss in mL (mean ± SD)		428 ± 98	320 ± 117	<0.001
	Vaginal delivery blood loss in mL (mean ± SD)		215 ± 88	
	Cesarean section blood loss in mL (mean ± SD)	428 ± 98	393 ± 71	0.2
Uterine atony/subatony (%)		0	0.36	0.8
Uterine rupture (%)		6.25	0.05	<0.001
HELLP (%)		6.25	0.09	<0.001
Fetal birth weight (g, mean ± SD)		1974 ± 984	3374 ± 918	<0.001
Live births (%)		100	99.18	0.7

## Data Availability

Data are available on request.
